# Alcohol-induced retrograde facilitation renders witnesses of crime less suggestible to misinformation

**DOI:** 10.1007/s00213-017-4564-2

**Published:** 2017-02-19

**Authors:** Julie Gawrylowicz, Anne M. Ridley, Ian P. Albery, Edit Barnoth, Jack Young

**Affiliations:** 1 Department of Psychology, School of Health and Life Sciences, Glasgow Caledonian University, Cowcaddens Road, Glasgow, Scotland G4 0BA UK; 2 Division of Psychology, School of Applied Sciences, London South Bank University, 103 Borough Road, London, SE1 0AA UK

**Keywords:** Eyewitness memory, Suggestibility, Alcohol, Retrograde facilitation, Interference

## Abstract

**Rationale:**

Research has shown that alcohol can have both detrimental and facilitating effects on memory: intoxication can lead to poor memory for information encoded *after* alcohol consumption (anterograde amnesia) and may improve memory for information encoded *before* consumption (retrograde facilitation). This study examined whether alcohol consumed *after* witnessing a crime can render individuals less vulnerable to misleading post-event information (misinformation).

**Method:**

Participants watched a simulated crime video. Thereafter, one third of participants expected and received alcohol (alcohol group), one third did not expect but received alcohol (reverse placebo), and one third did not expect nor receive alcohol (control). After alcohol consumption, participants were exposed to misinformation embedded in a written narrative about the crime. The following day, participants completed a cued-recall questionnaire about the event.

**Results:**

Control participants were more likely to report misinformation compared to the alcohol and reverse placebo group.

**Conclusion:**

The findings suggest that we may oversimplify the effect alcohol has on suggestibility and that sometimes alcohol can have beneficial effects on eyewitness memory by protecting against misleading post-event information.

According to the British Crime Survey (data from 2012/13 and 2013/14 combined), 70% of “public space” violent incidents were alcohol-related and 93% of those occurred in pubs, bars, and clubs where alcohol is sold and consumed (Office for National Statistics 2015). Evans et al. (2009) note that many witnesses are intoxicated either at the time of a crime, when interviewed or both. Given these numbers, studies have explored the effects of alcohol on various aspects of eyewitness memory including free recall, cued recall and line-up performance with generally detrimental (Crossland et al. 2016; Dysart et al. 2002; Van Oorsouw and Merckelbach 2012; Yuille and Tollestrup 1990) or null effects of alcohol consumption on memory (Hagsand et al. 2013; Harvey et al. 2013; Schreiber Compo et al. 2012). It should be noted that some of these studies obtained fairly low levels of intoxication with their participants and probably underestimated the detrimental effect of alcohol on memory performance. Amongst laboratory studies, the highest peak BAC was .20% (Dysart et al. 2002) and the smallest peak BAC was .09% (Hagsand et al. 2013). Low peak BACs ranged from .03% to .05% (Crossland et al. 2016; Harvey et al. 2013, respectively). In field studies by Van Oorsouw and Merckelbach (2012) and Van Oorsouw et al. (2015) slightly higher peak BACs of .24% and .26% were obtained. It could be argued that negative effects of alcohol on eyewitness memory performance might be even more pronounced when higher intoxication levels are present, however, due to ethical reasons this is often not achievable in laboratory studies. Recent research, for example by Crossland et al. (2016), Hagsand et al. (2013), Van Oorsouw and Merckelbach (2012) and Van Oorsouw et al. (2015) have managed to reach higher BAC levels, which should not be underestimated.

Only a few studies to date have examined the relationship between alcohol and suggestibility (Santtila et al. 1998, 1999; Schreiber Compo et al. 2012; Van Oorsouw et al. 2015). Suggestibility in eyewitnesses is the remembering or reporting of erroneous details about an event either in response to incorrectly leading questions (*immediate* suggestibility) or through exposure to incorrect information about the event that is later recalled (*delayed* suggestibility) (Ridley and Gudjonsson 2013; Schooler and Loftus 1993). For example, Van Oorsouw et al. (2015) approached sober (BACs <.02%; *M*_BAC_ = 0.01%, *SD* = 0.01), moderately intoxicated (BACs between .02% and .11%; *M*_BAC_ = 0.06%, *SD* = 0.02) or severely intoxicated (BACs > .11%; *M*_BAC_ = 0.16%, *SD* = 0.04) participants drinking in a bar and required them to commit a mock crime. In subsequent interviews (which included 15 incorrectly leading questions), those who were severely intoxicated were more suggestible to the leading questions than those who were sober. Conversely, Schreiber Compo et al. (2012), using a delayed rather than immediate suggestibility paradigm in a laboratory environment, found no difference between intoxicated (*M*_BAC_ = .07, *SD* = .02), placebo and control participants on the level of suggestibility. Importantly, in both studies, participants were intoxicated *before* witnessing the crime.

Santtila et al. (1998, 1999) explored the effect of alcohol on immediate suggestibility using the Gudjonsson Suggestibility Scale 2 (GSS2, Gudjonsson 1997). Participants received a high (1.32 ml of 95% alcohol per kg of body weight), medium (.66 ml of 95% alcohol per kg of body weight) or low dose of alcohol (.132 ml of 95% alcohol per kg of body weight), or a placebo drink. No BAC readings were taken after alcohol administration. Alcohol, when administered *after* the to-be-remembered narrative, appeared to protect participants from the negative effect of incorrectly leading questions. There was no effect when these questions were repeated following negative feedback. Santtila et al. (1998, 1999) argued that individual differences in emotions (e.g., anger and guilt) and personality (e.g., acquiescence and trait-anxiety) might be responsible for decreased suggestibility scores before negative feedback. However, personality and emotions did not moderate the effects of alcohol on suggestibility. These findings may reflect what Wixted (2004) called the “curious phenomenon of retrograde facilitation” (p. 254). Retrograde facilitation refers to memories being enhanced if they are formed prior to intoxication. One explanation for this twisted effect is that alcohol protects already formed memories by reducing new memory formation (Wixted 2005). That alcohol and benzodiazepines, if consumed prior to learning, can induce temporary anterograde amnesia to a point where individuals do not remember anything at all—alcoholic “blackout”—is well known (see Wetherill and Fromme 2016 for a review). However, these drugs may have a positive effect on memories formed prior to consumption by protecting them against any new incoming information, thereby reducing retrograde interference (Wixted 2005). It could be argued that this protective function may therefore lead to improved memory even in the absence of a direct interference task by reducing nonspecific interference and thereby protecting still fragile recently formed memories.

Evidence for this approach comes from several psychological experiments. For example, Parker et al. (1980) tested participants’ memory for scenic slides and found that having had alcohol (mean peak BAC = .08%) after learning significantly improved participants’ recognition performance. Similarly, Moulton et al. (2005) found that although memory for prose learned while participants were intoxicated (mean peak BAC = .08%) was poor compared to sober controls, the opposite was found for prose learned prior to intoxication. The diphasic effect of alcohol on memory performance has been found with emotional (Bruce and Pihl 1997; *M*_BAC_ = .06%), neutral, and alcohol-related stimuli (Weafer et al. 2016a, b; peak BAC = .08%). Thus, alcohol seems to impair subsequent recall of information encoded when intoxicated but facilitates recall of material encountered prior to intoxication due to minimizing general new memory formation (Wixted 2005). The diphasic effect of alcohol on memory performance could have important implications for forensic settings. That is, alcohol may not always render witnesses and victims to crime less reliable, but it depends on the timing of the incident and the alcohol consumption. For example, an individual might observe something in a club or pub prior to consuming any alcohol, and not realize that this will later form a crucial detail during a criminal investigation. Oblivious to the fact that he/she will become a witness, the individual might then consume alcohol during the remaining course of the evening. It could be argued that in such a scenario, this witness will be less susceptible to the incorporation of misinformation than an individual who remained sober.

The present study used a delayed misinformation paradigm to explore the effects of alcohol administered *after* a witnessed event but *prior* to exposure to misleading information. The misinformation paradigm is a popular task to study false memories and suggestibility and findings have important implications for situations such as eyewitness testimony and investigative interviewing. For instance, Loftus et al. (1978) found that information presented after an event can influence individuals’ memory for that event. In a series of experiments depicting an auto-pedestrian accident, participants who had initially seen a stop sign, later reported having seen a yield sign after having been exposed to misleading information. Since then, the misinformation effect has been examined with various stimuli and under a variety of conditions and researchers were even able to induce very rich false memories, such as having attended a hospital appointment (Ost et al. 2005) or committing a crime (Shaw and Porter 2015) (see Loftus 2005 for a review).

In the present experiment, alcohol was administered *after* witnessing a crime but *prior* to the presentation of misinformation. Cued recall was used to test suggestibility 24 h later when participants were sober again. To enable us to separate the physiological and psychological expectancy effects of alcohol on suggestibility a *reverse* placebo treatment condition (participants were told they would not receive alcohol when in fact they did) was included. This is fundamental given the significant body of work demonstrating that alcohol-outcome expectancies can influence different behaviors and cognitions (Moss and Albery 2009), such as impulsivity (Caswell et al. 2013), sexual risk taking (Maisto et al. 2012), self-perception (Bègue et al. 2013), motor performance (Fillmore and Vogel-Sprott 1995), and speed of processing (Fillmore et al. 1998), even in the absence of alcohol consumption.

## Method

### Participants and design

Eighty-three participants (students and staff from London South Bank University) (60 females, 23 males) aged 18 to 58 years (*M* = 27.38, *SD* = 9.45) took part in the study. One participant did not return to the second session. Participants either received course credits or a small monetary reward for their participation. Each individual completed a comprehensive screening process to establish their eligibility to participate. Age, weight, height, previous drinking history, and any medical conditions were established to ensure it was safe for them to consume alcohol. The experiment followed a 3 × 2 mixed design. The between-subjects factor was condition: alcohol, control, and reverse placebo. The within-subjects factor was item type: control vs. misled. That is all participants were exposed to misleading and control items and had to subsequently answer questions relating to these items. The study was approved by the London South Bank University’s Ethics Committee.

### Materials

#### Breath alcohol measurement

Breath alcohol content (BrAC) was measured with a Lion Alcolmeter 500 breathalyzer. This instrument was checked with an alcohol standard at regular intervals, and recalibrated if required. Participants’ BrAC was subsequently converted into BAC (blood alcohol contents) with the Lion Units Converter.

#### Crime event

The event was a video-recorded staged non-violent crime depicting a distractor burglary. The video footage showed two people, a man and a woman, entering a house and conducting a survey with the homeowner in the living room. After a few minutes, the homeowner went into the kitchen to make some tea. In the mean time, the woman went upstairs into a room and stole some jewelry, while the man stole some money and a laptop from the living room. The robbers then swiftly left the property. The homeowner retuned from the kitchen and ran after the thieves after he realized that he has been robbed. The event was 4 min and 25 s long.

#### Post-event information (PEI) and memory test

Pilot testing was conducted to ensure that memory for each critical detail was reasonable and the misleading suggestions were seen as plausible. Twenty-nine participants, comprising of University students and staff (*M*_age_ = 30.06, *SD* = 9.11, 16 females), watched the video clip and then completed a cued recall test immediately. Only items that were remembered more often than 40% and less than 90% of the time were selected as potential critical items. Next, participants were presented with a multiple-choice test including the correct answer to the previously presented cued recall test and three alternative answers. Participants were asked to circle the option that was actually presented in the video clip and to rate the plausibility of each other alternative on a 1 (highly implausible) to 7 (highly plausible) scale. Items with mean plausibility ratings of 2–6 were included as the misleading details. This was done to ensure that misinformation items were not completely implausible and therefore directly rejected by participants. Similarly, it was important to ensure that misinformation items were not too plausible and therefore more likely to be accepted by participants than original items (a similar procedure was used by Horry et al. 2014). The mean plausibility rating for misleading items was 4.14 (SD = 1.34). After pilot testing, 10 items (out of 30) were selected as critical items.

The PEI was presented in a 330-word narrative. Two versions of the narrative were created (versions A and B). Each version included five misleading items. The five misleading items for half of the participants served as control items for the other half of participants in order to give an indication of the extent to which PEI is recounted by chance. Most of the control items were not mentioned in the narrative or were mentioned only in a neutral form (as in Horry et al. 2014). For example, in version A, participants read the misinformation that the victim was wearing a *green* fleece jumper (misleading item), whereas in version B the victim was only described as wearing a fleece jumper (control item), in the original video footage the jumper was *blue*.

The cued recall memory test consisted of 10 questions—five enquiring about misleading items and five about control items. For example, “What was the colour of the victims jumper?” or “What did the female perpetrator carry?”. Underneath each question was some space for participants to provide their answer. Underneath the space for the answer was a confidence rating scale ranging from 0% (not very confident at all) to 100% (very confident).

### Procedure

The study consisted of two sessions separated by one day. Participants took part individually. During the first session, participants watched the staged crime video. They were told to pay close attention. Subsequently, participants were randomly assigned to one of three conditions—received alcohol (Beck’s lager) and expected alcohol (alcohol condition, *N* = 28), received alcohol (Beck’s lager) and did not expect alcohol (participants were told they received Beck’s Blue, an alcohol-free lager) (reverse placebo condition, *N* = 28) and received no alcohol (Beck’s Blue) and expected no alcohol (control condition, *N* = 27). Participants’ gender, height, and weight were used to calculate the required dose with the Curtin’s Blood Alcohol Calculator to achieve a peak BAC of approximately 0.06% (Curtin 2000). All drinks were poured out of sight of the participants. On being handed their beverage, participants were told to consume their drink within 30 min but no faster than 20 min (a similar procedure was used in Santtila et al. 1998, 1999). Participants then rinsed their mouth with water to remove residual traces of alcohol and to avoid contamination of subsequent breathalyzer readings. Thereafter, participants completed some filler tasks (word search games) for 15 min to allow their BAC to rise (see Crossland et al. 2016). Participants were then breathalyzed (but not informed about the reading). If their BAC reading was below the required 0.06% a further reading was taken 15 min later. It is important to note that all participants underwent the same procedure regardless of condition. That is, all participants had to consume the beverage in the above-described time and all participants were subsequently breathalyzed after a 15-min delay at least once.^1^

All participants were then presented with one of two versions of the post-event narrative. To ensure that the information was being processed, the narrative was presented on six different cards in a randomly scrambled order. Participants were told that they would engage in a comprehension task and that they had to read the cards carefully and subsequently sort them in chronological order (this procedure was adopted from Horry et al. 2014). Finally, participants were asked to read out aloud the sorted narrative cards to the experimenter. Following the comprehension task, participants were informed about the actual nature of their drink and released from the first session. Those displaying BACs above .08% (the UK drink-drive limit) were advised to remain in the lab until their BAC reduced to below 08%. Those who did not wish to stay behind had to sign a waiver form, confirming their awareness of being in excess of the UK drink and drive alcohol limit.

The second session commenced the following day. All participants completed the cued-recall memory test in their own time. They were instructed to think back to the film they watched and for every question to write down what they remember having seen in the film. They were told that the narrative they read during the comprehension task contained some incorrect details. They were instructed to provide a response to every question. If they could not remember, they were asked to guess. After each question, participants were asked to rate the confidence in their answer on a scale ranging from 0% (not confident at all) to 100% (very confident). After having answered all questions, participants were asked to go back through their answers and to indicate for each whether they would be willing to testify to it in a court of law. With this in mind, participants went through their answers and indicated with a “T” next to the answer that they would testify to it or with a “W” next to it that they would withhold the answer (e.g., Horry et al. 2014). Finally, participants were debriefed, thanked for their participation, and dismissed.

## Results

### Blood alcohol concentrations

In line with previous research, all BrACs were converted to BACs with the blood: breath ratio of 2300:1. For the two groups who consumed alcohol (i.e., alcohol and reverse placebo group), the average BAC was .065%; BACs ranged from .03% to .11%. All control participants had a BAC of .00%. A one-way ANOVA revealed a significant difference between conditions, *F* (2, 80) = 174.34, *p* < .001, *η* ^2^ = 0.82. The control group differed significantly from both the alcohol and reverse placebo group (*p*s < .001). There was no significant difference between the alcohol and the reverse placebo group (*p* > .999) (alcohol group: *M* = .063, *SD* = .02, 95% CI = [.056, .070]; reverse placebo group: *M* = .067, *SD* = .02, 95% CI = [.060, .073]).

### Coding and analysis of memory test data

Responses were coded as correct, misled (i.e., contained misinformation provided in narrative), or incorrect (i.e., containing other incorrect information). For example, a response was coded as correct when it coincided with what was actually shown in the video footage, i.e., the victim wore a *blue* fleece jumper in the video and the participant reported on the cued recall test that the fleece jumper was *blue*. A response was coded as misled when it coincided with the misinformation presented in the narrative and not with the information provided in the video clip, i.e., the narrative mentioned that the victim wore a *green* fleece jumper, whereas it was *blue* in the video clip, and the participant reported on the cued recall test that it was *green*. A response was coded as incorrect, when it did not coincide with either the information provided in the video footage or the narrative, i.e., the participant reported that the fleece jumper was *red*. To assess inter-coder reliability, 20% of the cued recall memory tests (16 tests) were coded by two independent coders. Significant Pearson correlations between coders were established (correct responses: *r* = .991, *p* < .001; misled responses: *r* = 1, *p* < .001; incorrect responses: *r* = .989, *p* < .001).

### Proportion of misled responses

A 2 (item: control vs. misled) × 3 (Condition: alcohol vs. control vs. reverse placebo) mixed ANOVA on the proportion of misled responses (out of five) revealed a significant main effect for item, *F* (1, 79) = 35.39, *p* < .001, *η* ^2^ = .0.189. Participants were more likely to provide a misled response to misinformation items (*M* = .36, 95%CI [.309, .405]) than to control items (*M* = .17, 95%CI [.133, .209]). The main effect for condition was also significant, *F* (2, 79) = 3.51, *p* = .035, *η* ^2^ = .088). Bonferroni post-hoc tests showed that the control group provided significantly more misled responses than the reverse placebo group (*p* = .038). There was no significant difference between the control and alcohol group (*p* = .183) nor between the reverse placebo and alcohol group (*p* > .999) (alcohol: *M* = .25, 95%CI [.196, .300]; control: *M* = .319, 95%CI [.266, .371]; reverse placebo: *M* = .22, 95%CI [.174, .276]). The interaction between item and condition was significant, *F* (2, 79) = 8.55, *p* < .001, *η* ^2^ = .178. Bonferroni post-hoc tests revealed that groups did not differ significantly on control items (alcohol vs. control: *p* = .478; alcohol vs. reverse placebo: *p* > .999; reverse placebo vs. control: *p* > .999). However, for the misinformation items a significant difference was present between the alcohol and control group (*p* = .002), and between the reverse placebo and control group (*p* = .001), but not between the alcohol and reverse placebo group (*p* > .999). Thus, the control group was significantly more likely to give a misled response to misleading items compared to the alcohol and reverse placebo group.^2^

### Proportion correct responses

A 2 (item: control vs. misled) × 3 (condition: alcohol vs. control vs. reverse placebo) mixed ANOVA on the proportion of correct responses revealed a significant main effect for item, *F* (1, 79) = 8.84, *p* = .004, η^2^ = .101. Participants provided significantly more correct responses to control items (*M* = .55, 95%CI [.498, .609]) than to misleading items (*M* = .44, 95%CI [.384, .498]). The main effect for condition, *F* (2, 79) = .05, *p* = .956, η^2^ = .001, and the interaction between item and condition were not significant, *F*(2, 79) = 1.34, *p* = .267, *η* ^2^ = .033. Proportions correct and misled responses are presented in Table 1.

**Table 1:** Mean (and 95% CI) proportion correct (out of 5) and misled (out of 5) responses for control and misleading items in each condition (alcohol, control and reverse placebo)

Item	Condition					
	Alcohol		Control		Reverse Placebo	
	Control	Misleading	Control	Misleading	Control	Misleading
Correct responses	.52 [.422, .615]^a^	.46 [.360, .558]^a^	.60 [.503, .697]^b^	.40 [.301, .499]^b^	.54 [.448, .638]^c^	.46 [.367, .561]^c^
Misled responses	.20 [.134, .266]	.30 [.213, .380]^d^	.13 [.067, .199]	.50 [.420, .587]^de^	.17 [.114, .243]	.27 [.189, .353]^e^

^a b c d e^Cells sharing the same capital differ significantly from each other

### Proportion testified responses

A 3 (response type: correct, incorrect, misled) × 3 (condition: alcohol vs. control vs. reverse placebo) mixed ANOVA on the proportion of testified responses revealed a significant main effect for response type, *F* (2, 158) = 89.84, *p* < .001, *η* ^2^ = .532 (correct: *M* = .50, 95%CI [.456, .539]; incorrect: *M* = .08, 95%CI [.056, .105]; misled: *M* = .36, 95%CI [ .309, .405]). The main effect for condition was also significant, *F* (2, 79) = 6.50, *p* = .002, *η* ^*2*^ = .141 (alcohol: *M* = .30, 95%CI [.271, .322]; control: *M* = .35, 95%CI [.324, .375]; reverse placebo: *M* = .29, 95%CI [.264, .315]). In addition, the interaction between response type and condition was significant *F* (4, 158) = 4.66, *p* = .001, *η* ^2^ = .106. Bonferroni post-hoc tests showed that there were no group differences in the proportion of testified responses for correct and incorrect responses (all *p*s > .05). However, the control group was significantly more likely to testify to a misled response compared to the alcohol (*p* = .002) and the reverse placebo group (*p* = .001). There was no significant difference between the alcohol and the reverse placebo group (*p* > .999). See Table 2 for mean proportions of testified responses and confidence intervals.

**Table 2:** Mean proportion of testified responses (and 95% confidence intervals) for correct (out of 10), incorrect (out of 10) and misled (out of 5) responses for each condition (alcohol, control and reverse placebo)

Proportion testified	Condition		
	Alcohol	Control	Reverse Placebo
Correct responses	.49 [.416, .561]	.50 [.427, .573]	.50 [.432, .575]
Incorrect responses	.10 [.061, .146]	.04 [.002, .087]	.09 [.051, .135]
Misled responses	.30 [.213, .380]^a^	.50 [.420, .587]^ab^	.27 [.189, .353]^b^

^a b^Cells sharing the same capital differ significantly from each other

### Confidence ratings in correct, incorrect and misled responses

A 3 (response: correct vs. incorrect vs. misled) × 3 (Condition: alcohol vs. control vs. reverse placebo) mixed ANOVA revealed a significant main effect for Response Type, *F* (2, 132) = 79.39, *p* < .001, *η* ^2^ = .546 . Bonferroni post-hoc tests revealed that participants provided significantly lower confidence ratings for errors compared to misled (*p* < .001) and correct responses (*p* < .001). Confidence ratings for misled responses were also significantly lower than for correct responses (*p* = .003). Neither the main effect for Condition, *F* (2, 66) = .58, *p* = .562, *η* ^2^ = .017, nor the interaction between Response Type and Condition, *F* (2, 66) = 1.03, *p* = .390, *η* ^2^ = .031 was significant. See Table 3 for mean confidence ratings and confidence intervals.

**Table 3:** Mean (95% confidence intervals) of confidence ratings to correct, incorrect and misled responses for each condition (alcohol, control and reverse placebo)

Confidence %	Condition		
	Alcohol	Control	Reverse placebo
Correct responses	74.30 [66.61, 81.98]^a^	70.64 [62.63, 78.66]^b^	73.20 [64.81, 81.58]^c^
Incorrect responses	33.80 [23.43, 44.16]^a^	37.25 [26.44, 48.05]^b^	39.56 [27.79, 50.41]^c^
Misled responses	57.13 [48.76, 65.51]^a^	65.14 [56.41, 73.87]^b^	69.29 [60.15, 78.42]^c^
Testified responses	78.41 [72.36, 84.47]^d^	79.19 [73.25, 85.13]^e^	80.86 [74.93, 86.80]^f^
Withheld responses	35.85 [28.92, 42.78]^d^	39.99 [33.19, 46.79]^e^	38.07 [31.27, 44.87]^f^

^a b c d e f^Cells sharing the same capital differ significantly from each other

### Confidence ratings in testified and withheld responses

A 2 (response type: testify vs. withheld) × 3 (condition: alcohol vs. control vs. reverse placebo) mixed ANOVA on the mean confidence ratings for testified and withheld responses revealed a significant main effect for response type, *F* (1, 77) = 308.43, *p* < .001, *η* ^2^ = .800. Participants were significantly more confident in testified responses (*M* = 79.50, 95%CI [76.04, 82.94]) than in withheld responses (*M* = 37.99, 95%CI [34.02, 41.92]). No significant main effect for condition was revealed, *F* (2, 77) = .30, *p* = .738, *η* ^2^ = .008. The interaction between response type and condition was not significant, *F* (2, 77) = .24, *p* = .785, *η* ^2^ = .006. See Table 3 for mean confidence ratings and 95% confidence intervals.

## Discussion

The current study was designed to examine alcohol-induced retrograde facilitation in a delayed misinformation paradigm. We are the first to show that alcohol consumption after having witnessed a criminal event can protect memory from the negative effects of misinformation. Contrary to the general belief and empirical finding that alcohol impairs eyewitness memory, we found that individuals who consumed alcohol (alcohol and reverse placebo group) *after* the observed event but *prior* to encountering misleading information, reported significantly fewer misinformation items on a subsequent memory test compared to the control group (who did not receive any alcohol). A particularly worrying finding is that the sober participants were also more likely to be prepared to testify a misled response in a court of law. No difference in the proportion of correct responses provided was found between groups. Nor did the groups differ with regards to how confident they were in their responses.

A potential explanation for reduced suggestibility is that alcohol leads to decreased retrograde interference (Mueller et al. 1983). That is intoxication reduces the formation of new memories thereby protecting already existing memories and making them less vulnerable to interference. Alcohol-induced retrograde facilitation has been demonstrated in previous studies using list of words, sentences, pictures and prose passages (Bruce and Pihl 1997; Moulton et al. 2005; Parker et al. 1980; Weafer et al. 2016a, b). Our findings demonstrate that alcohol-induced facilitation holds true for autobiographical memories in a misinformation eyewitness paradigm.

Including a reverse placebo group allowed us to shed more light on expectancy and pharmacological effects of alcohol consumption on retrograde facilitation. Both the alcohol and reverse placebo group were less suggestible to misinformation, indicating that the facilitated memory effect is more likely to be due to the pharmacological effects of alcohol rather than due to alcohol-related beliefs. This is in line with the findings reported by Parker et al. (1981) who showed that the alcohol-induced facilitation effect is dose-dependent. These are tentative conclusions and further research is needed to disentangle expectancy and pharmacological effects on retrograde memory facilitation. The current study did not measure participants’ subjective beliefs regarding their intoxication level, and we cannot preclude that control participants thought that they were actually drinking alcohol (even if they were told that they received Beck’s Blue).

Furthermore, our results disconfirm a competing theory for enhanced learning prior to alcohol consumption—improved consolidation of memory traces (Parker et al. 1980). According to this theory, alcohol increases processing (i.e., consolidation) of previously learned material. Contrary to this view, we did not find group differences in the proportion of correct responses provided. Our findings are more in line with the interference-reduction view (Mueller et al. 1983). That is, alcohol reduces the amount of new incoming information from entering memory and as a result preserves already existing memories. Intoxicated eyewitnesses were consequently less suggestible to misinformation in our study.

It is important to note that we are not implying that incoming misinformation impairs memory for the original event in the absence of alcohol. As highlighted and demonstrated by McCloskey and Zaragoza (1985) in a series of studies, originally formed memories may still be intact and co-exist together with the misleading information. They used the misinformation paradigm but included a modified test version. Depending on their condition, participants were either presented with misinformation or no misinformation (control group). At test, participants were presented with two forced-choice alternatives, the original item and a new item (the misleading information was not an option). They found no difference in performance between groups at test. That is, misled participants were as likely as control participants to choose the original item, providing no evidence for memory impairment.

To test whether alcohol indeed hinders new information from entering memory, future research should use a forced-choice recognition test, similar to McCloskey and Zaragoza’s (1985) modified test version, rather than a cued recall test. The test alternatives would be the misinformation item and a new item. If alcohol really blocks/reduces interfering information from entering memory, then participants in the alcohol group should be as likely to choose the misinformation as the new item at test (chance performance). Control participants, however, should be more likely to pick the misinformation item compared to the new item. This finding would provide independent evidence for the claim that alcohol blocks/reduces misinformation from entering memory.

In relation to the confidence ratings given for cued-recall responses, we did not find any differences between groups. However, we did find that mean confidence levels differed across response type. Participants were significantly more confident in correct responses compared to misled and incorrect responses. Intriguingly, participants were also significantly more confident in misled responses than in incorrect ones. The source-monitoring framework (Johnson et al. 1993) may offer an explanation for this finding. When participants are asked a cued recall question, several response alternatives are generated. The participant has to then decide whether to report one or not. This decision is dependent on whether the participant can retrieve any source memory for the alternative answers (i.e., any spatial, temporal, modal, or social contextual cues). Erroneous responses are usually associated with an inability to specify the source of the information and are therefore reported with low confidence. Individuals might report suggested details with higher confidence because either the misled response might wrongly elicit event-related cues (i.e., source confusion error), or the misled response was retrieved easily (i.e., with high retrieval fluency). In both cases, the misled response would be given with increased confidence (Horry et al. 2014). It is interesting to note that in the current study although participants were on average more confident in misled responses than in errors, they were most confident in correct responses.

It remains to be seen whether the alcohol-induced retrograde facilitation effect can be generalized to other types of misinformation. Misinformation can be introduced indirectly, through embedding it in a set of questions (Loftus et al. 1978), or directly, through a face-to-face encounter (Blank et al. 2013; Gabbert et al. 2003). The scrambled sentence task used in this study can be regarded as an indirect way of introducing misinformation. Further research will be necessary to explore the impact of alcohol on suggestibility when the misinformation is presented in a more direct way.

Future research should also explore whether alcohol-induced retrograde facilitation is present in more realistic forensic settings. Only a few field studies on the impact of alcohol on eyewitness memory performance exist (Van Oorsouw and Merckelbach 2012; Van Oorsouw et al. 2015) and none has explored retrograde facilitation. For example, is this phenomenon also present when people are mildly intoxicated when witnessing the event and more severely intoxicated when presented with misinformation? Such a scenario could be regarded as more ecologically valid, as witnesses may be already mildly intoxicated before observing a crime. The average peak BAC of .065% in the current study could be considered fairly low, and future research should test alcohol-induced retrograde facilitation under higher levels of intoxication, which are more likely to be found in the real world. From a practical and theoretical perspective, it would be also valuable to examine how long the protective effect of alcohol against misinformation lasts for. There are often lengthy passages of time between the crime and the recalling of the event by the witness. It could be argued that with longer delays, all individuals might become more suggestible to misinformation regardless of their intoxication level *after* encoding and *prior* to the endorsement of misinformation (e.g., Hertel et al. 1980; Loftus et al. 1978). Would the inoculating effect of alcohol against misinformation disappear with longer time delays? Future studies should also examine the effects of alcohol on retrograde facilitation on different limbs (ascending and descending) of the BAC curve. Research has shown that alcohol might have differential effects on memory depending on the task, the memory type, and limb of the BAC curve (Söderlund et al. 2005).

In summary, our findings indicate that the timing of alcohol consumption plays an important role in determining how accurate and reliable subsequent memory reports are. The assumption that alcohol inevitably impairs eyewitness memory performance is oversimplified. We suggest that in certain circumstances alcohol intoxication can protect eyewitness memory by making individuals less suggestible to misinformation. Scientists who are conducting research in the field need to take this into account when designing their experiments and interpreting their findings, for example by asking participants about subsequent drinking behavior between testing sessions.

## Footnotes

1 Given that some individuals were breathalyzed twice, the delay between watching the crime video and the subsequent presentation of misinformation varied by approximately15 min.

2 A 2 × 3 between-participants ANOVA was conducted on the number of misled responses including the Narrative Version (A vs. B) as another independent variable. A significant main effect for Narrative version (*F* (1, 72) = 10.34, *p* = .002) and condition (*F* (2, 76) = 10.03, *p* < .001, *η* ^2^ = .209) was revealed. Participants gave more misled responses for narrative A (*M* = 2.14, 95%CI [1.825, 2.460]) compared to narrative B (*M* = 1.40, 95%CI [1.083, 1.734]). Post-hoc tests showed that the control group provided significantly more misled responses than the alcohol (*p* = .001) and the reserve placebo groups (*p* < .001). There was no significant difference for misled responses between the alcohol and reverse placebo group (*p* > .999). Importantly, the interaction between narrative version and condition was not significant (*F* (2, 76) = .83, *p* = .441, η^2^ = 0.21). Thus, individuals gave more misled responses when provided with narrative A compared to narrative B. However, this effect did not moderate the effect of Condition.
